# Chebyshev type inequalities by means of copulas

**DOI:** 10.1186/s13660-017-1549-y

**Published:** 2017-10-30

**Authors:** Sever S Dragomir, Eder Kikianty

**Affiliations:** 1 College of Engineering and Science, Victoria University, PO Box 14428, Melbourne, VIC 8001 Australia; 2 DST-NRF Centre of Excellence in the Mathematical and Statistical Sciences, School of Computer Science and Applied Mathematics, University of the Witwatersrand, Private Bag 3, Wits, Johannesburg, 2050 South Africa; 3 Department of Mathematics and Applied Mathematics, University of Pretoria, Private Bag X20, Hatfield, 0028 South Africa

**Keywords:** Chebyshev inequality, synchronous function, copula, *t*-norm

## Abstract

A copula is a function which joins (or ‘couples’) a bivariate distribution function to its marginal (one-dimensional) distribution functions. In this paper, we obtain Chebyshev type inequalities by utilising copulas.

## Introduction

A copula is a function which joins (or ‘couples’) a bivariate distribution function to its marginal (one-dimensional) distribution functions. Mathematically defined, a copula *C* is a function $C:[0,1]^{2}\to[0,1]$ with the following properties:

1. $C(u,0)=C(0,u)=0$, $C(u,1)=u$, and $C(1,u)=u$ for all $u\in[0,1]$,
2. $C(u_{1},v_{1})-C(u_{1},v_{2})-C(u_{2},v_{1})+C(u_{2},v_{2})\geq0$ for every $u_{1},u_{1},v_{1},v_{2} \in[0,1]$ such that $u_{1} \leq u_{2}$ and $v_{1} \leq v_{2}$.

Property (C2) is referred to as the 2-increasing property, or moderate growth [1]. The 2-increasing property implies the following properties for any copula *C*:

(C4) *C* is nondecreasing in each variable;
(C5) *C* satisfies the Lipschitz condition: for all $u_{1},u_{2},v_{1},v _{2}\in[0,1]$,
  $$\bigl\vert C(u_{2},v_{2})-C(u_{1},v_{1}) \bigr\vert \leq \vert u_{2}-u_{1}\vert +\vert v_{2}-v_{1}\vert . $$

For further reading on copulas, we refer the readers to [2] and [3].

While copulas join probability distributions, *t*-norms join membership functions of fuzzy sets, and hence combining probabilistic information and combining fuzzy information are not so different [4]. Mathematically defined, a *t*-norm *T* is a function $T:[0,1]^{2}\rightarrow [0,1]$ with the properties [4]:

1. Commutativity: $T(x,y)=T(y,x)$ for all $x,y\in[0,1]$,
2. Associativity: $T(x,T(y,z))=T(T(x,y),z)$ for all $x,y,z\in[0,1]$,
3. Monotonicity: $T(x,y)\leq T(x,z)$ for all $x,y,z\in[0,1]$ with $y\leq z$,
4. Boundary condition: $T(x,1)=T(1,x)=x$, $T(x,0)=T(0,x)=x$ for all $x\in[0,1]$.

A copula is a *t*-norm if and only if it is associative; conversely, a *t*-norm is a copula if and only if it is 1-Lipschitz [1]. The three main continuous *t*-norms, namely the minimum operator ($M(x,y)= \min\{x,y\}$), the algebraic product ($P(x,y)=xy$), and the Lukasiewicz *t*-norm ($W(x,y)=\max\{x+y-1,0\}$), are copulas.

The first importance of these copulas is given by the following: Let *C* be a copula, then

1.1 $$ W(u,v)\leq C(u,v) \leq M(u,v)\quad \mbox{for all }u,v\in[0,1]. $$

The above inequality is referred to as the Fréchet-Hoeffding bounds for copulas and provides a basic inequality for copulas. Inequality (1.1) also holds in the contexts of probability theory and fuzzy probability calculus [1] and is referred to as the Bell inequalities. Further inequalities for copulas of Bell type are given in [1]. Other inequalities for copulas are given in [5] in relation to a family of continuous functions *L* from $[0,\infty]\times[0,\infty]$ onto $[0,\infty]$ which are nondecreasing in each variable with $\lim_{x\rightarrow\infty}L(x,x)= \infty$.

Egozcue et al. [6] established Grüss type bounds for covariances by assuming the dependence structures such as quadrant dependence and quadrant dependence in expectation. They utilise copulas to illustrate these dependent structures.

In the same spirit to [6], it is our aim here to establish inequalities by utilising copulas. Firstly, we note the connection between the 2-increasing property and the Chebyshevian mappings. A mapping $F:[a,b]^{2}\rightarrow\mathbb{R}$ is called *Chebyshevian* on $[a,b]^{2}$ if the following inequality is satisfied:

$$F(x,x)+F(y,y)\geq F(x,y)+F(y,x) \quad\mbox{for all } x,y \in[a,b]. $$

Let *C* be a copula, $x,y\in[0,1]$, and set $u_{1}=u_{2}=x$ and $v_{1}=v_{2}=y$ in property (C2) (2-increasing property) above to obtain $C(x,x)-C(x,y)-C(y,x)+C(y,y)\geq0$, or equivalently,

1.2 $$ C(x,x)+C(y,y)\geq C(x,y)+C(y,x), $$

i.e. *C* is Chebyshevian on $[0,1]^{2}$.

Dragomir and Crstici [7] established the relationship between two synchronous functions and Chebyshevian mappings. Two functions $f,g:[a,b]\rightarrow\mathbb{R}$ are *synchronous* on $[a,b]$ if they have the same monotonicity, that is,

$$\bigl(f(x)-f(y)\bigr) \bigl(g(x)-g(y)\bigr)\geq0 \quad\mbox{for all }x,y\in[a,b]. $$

The relationship between the two notions is given in the following result.

### Proposition 1

(Dragomir and Crstici [7])

*If* *f*, *g* *are synchronous on* $[a,b]$ *and* $F:[a,b]^{2}\rightarrow \mathbb{R}$, *where* $F(x,y)=f(x)g(y)$, *then* *F* *is Chebyshevian on* $[a,b]^{2}$.

Consequently, the following Chebyshev type inequalities can be stated (see also Lehmann [8, Lemma 2]).

### Proposition 2

(Dragomir and Crstici [7])

*Let* $p:[a,b]\rightarrow\mathbb{R}$ *be integrable and nonnegative on* $[a,b]$.

1. *Let* $F:[a,b]^{2}\rightarrow\mathbb{R}$. *If* *F* *is Chebyshevian on* $[a,b]^{2}$, *then*
  1.3 $$ \int_{a}^{t}p(x)\,dx \int_{a}^{t} p(x)F(x,x)\,dx\geq \int_{a}^{t} \int_{a}^{t}p(x)p(y)F(x,y)\,dx \,dy $$
  *for all* $t\in[a,b]$.
2. *Let* $f,g:[a,b]\rightarrow\mathbb{R}$ *be integrable on* $[a,b]$. *If* *f* *and* *g* *are synchronous on* $[a,b]$, *then we have Chebyshev’s inequality*
  1.4 $$ \int_{a}^{t}p(x)\,dx \int_{a}^{t} p(x)f(x)g(x)\,dx\geq \int_{a}^{t}p(x)f(x)\,dx \int_{a}^{t}p(x)g(x)\,dx $$
  *for all* $t\in[a,b]$.

If $f,g:[a,b]\rightarrow[0,1]$ are synchronous, then by Proposition 1, the product copula given by

$$P\bigl(f(x),g(y)\bigr)=f(x)g(y),\quad x,y\in[a,b] $$

is Chebyshevian on $[a,b]^{2}$, as a consequence of the 2-increasing property. If we define a function $F:[a,b]^{2}\rightarrow[0,1]$ by $F(x,y)=P(f(x),g(y))=f(x)g(y)$, and if $p:[a,b]\rightarrow\mathbb{R}$ is integrable and nonnegative, then Proposition 2 gives us

$$\int_{a}^{t}p(x)\,dx \int_{a}^{t} p(x)P\bigl(f(x),g(x)\bigr)\,dx\geq \int_{a} ^{t} \int_{a}^{t}p(x)p(y)P\bigl(f(x),g(y)\bigr)\,dx \,dy. $$

Motivated by this observation, we aim to obtain other types of Chebyshev inequalities by utilising (the general definition of) copulas instead of the product copula as demonstrated above. Specifically, we provide inequalities for the dispersion of a function *f* defined on a measure space $(\Omega, \Sigma, \mu)$, with respect to a positive weight *ω* on Ω with $\int_{\Omega}\omega(t)\,d\mu(t)=1$, that is,

$$\biggl( \int_{\Omega}\omega f^{2}\,d\mu- \biggl( \int_{\Omega}\omega f \,d\mu\biggr) ^{2} \biggr) ^{\frac{1}{2}}. $$

## Chebyshev type inequalities

The 2-increasing property of copulas gives us the following result.

### Proposition 3

*Let* $C:[0,1]^{2}\rightarrow[0,1]$ *be a copula and* $p:[0,1]\rightarrow \mathbb{R}$ *be an integrable function*. *Then*

1. *C* *is Chebyshevian on* $[0,1]^{2}$.
2. *If* *p* *is nonnegative*, *then*
  $$\int_{0}^{t}p(x)\,dx \int_{0}^{t}p(x)C(x,x)\,dx \geq \int_{0}^{t} \int_{0}^{t} p(x)p(y)C(x,y)\,dx \,dy. $$

Proof follows by (1.2) and Proposition 2 part 1.

Now we state a more general form of this inequality. We start with the following lemma.

### Lemma 1

*Let* $f,g:[a,b]\rightarrow[0,1]$ *be two synchronous functions and* *C* *be a copula*. *Then* $F:[a,b]^{2}\rightarrow[0,1]$ *defined by*

$$F(x,y):=C\bigl(f(x),g(y)\bigr) $$

*is Chebyshevian on* $[a,b]^{2}$.

### Proof

Since *f* and *g* are synchronous, they have the same monotonicity on $[a,b]$. Let *A* be the collection of subsets of $[a,b]$ where *f* and *g* are both nondecreasing. Suppose that $x,y\in A$. Without loss of generality, let $x\leq y$, and set

$$u_{1}=f(x),\qquad u_{2}=f(y),\qquad v_{1}=g(x),\qquad v_{2}=g(y). $$

Thus, $u_{1} \leq u_{2}$ and $v_{1} \leq v_{2}$ since *f* and *g* are nondecreasing. Therefore, the 2-increasing property of *C* gives

$$\begin{aligned} 0 \leq&C(u_{1},v_{1})-C(u_{1},v_{2}) -C(u_{2},v_{1})+ C(u_{2},v_{2}) \\ =&C\bigl(f(x),g(x)\bigr)-C\bigl(f(x),g(y)\bigr) -C\bigl(f(y),g(x)\bigr)+ C \bigl(f(y),g(y)\bigr) \\ =&F(x,x)-F(x,y) -F(y,x)+ F(y,y). \end{aligned}$$

Suppose that $x,y\in[a,b]\setminus A$. Without loss of generality, let $x\leq y$,

$$u_{1}=f(y),\qquad u_{2}=f(x),\qquad v_{1}=g(y),\qquad v_{2}=g(x). $$

Thus, $u_{1} \leq u_{2}$ and $v_{1} \leq v_{2}$ since *f* and *g* are decreasing. Therefore, the 2-increasing property of *C* gives

$$\begin{aligned} 0 \leq&C(u_{1},v_{1})-C(u_{1},v_{2}) -C(u_{2},v_{1})+ C(u_{2},v_{2}) \\ =&C\bigl(f(y),g(y)\bigr)-C\bigl(f(y),g(x)\bigr) -C\bigl(f(x),g(y)\bigr)+ C \bigl(f(x),g(x)\bigr) \\ =&F(y,y)-F(y,x) -F(x,y)+ F(x,x). \end{aligned}$$

We show that *F* is Chebyshevian in both cases. □

Lemma 1 and Proposition 2 part 1 give us the following.

### Theorem 1

*Let* *C* *be a copula*, $f,g:[a,b]\rightarrow[0,1]$ *be two synchronous functions*, $p:[a,b]\rightarrow\mathbb{R}$ *be an integrable function*. *If* *p* *is nonnegative*, *then*

$$\begin{aligned}& \int_{a}^{t}p(x)\,dx \int_{a}^{t}p(x)C\bigl(f(x),g(x)\bigr)\,dx \\& \quad \geq \int_{a} ^{t} \int_{a}^{t} p(x)p(y)C\bigl(f(x),g(y)\bigr)\,dx \,dy. \end{aligned}$$

### Example 1

In this example, we obtain some Chebyshev type inequalities by choosing some examples of copulas. Let $f,g:[a,b]\rightarrow[0,1]$ be two synchronous functions, and $p:[a,b]\rightarrow\mathbb{R}$ be a nonnegative integrable function. Theorem 1 and (1.1) give us the following inequalities:

2.1 $$\begin{aligned}& \int_{a}^{t} \int_{a}^{t} p(x)p(y)\max\bigl\{ f(x)+g(y)-1,0\bigr\} \,dx \,dy \\& \quad \leq \int_{a}^{t}p(x)\,dx \int_{a}^{t}p(x)\max\bigl\{ f(x)+g(x)-1,0\bigr\} \,dx \\& \quad \leq \int_{a}^{t}p(x)\,dx \int_{a}^{t} p(x) C\bigl(f(x),g(x)\bigr)\,dx \\& \quad \leq \int_{a}^{t}p(x)\,dx \int_{a}^{t} p(x)\min\bigl\{ f(x),g(x)\bigr\} \,dx. \end{aligned}$$

The first inequality follows from Theorem 1 (by choosing the *W* copula) and the rest follows from the Fréchet-Hoeffding bound (1.1). Similarly, we have

2.2 $$\begin{aligned}& \int_{a}^{t} \int_{a}^{t} p(x)p(y) \max\bigl\{ f(x)+g(y)-1,0\bigr\} \,dx \,dy \\& \quad \leq \int_{a}^{t} \int_{a}^{t} p(x)p(y) C\bigl(f(x),g(y)\bigr)\,dx \,dy \\& \quad \leq \int_{a}^{t} \int_{a}^{t} p(x)p(y)\min\bigl\{ f(x),g(y)\bigr\} \,dx \,dy \\& \quad \leq \int_{a}^{t}p(x)\,dx \int_{a}^{t}p(x)\min\bigl\{ f(x),g(x)\bigr\} \,dx. \end{aligned}$$

The last inequality follows from Theorem 1 (by choosing the *M* copula), and the rest follows from the Fréchet-Hoeffding bounds (1.1).

In what follows, we generalise Theorem 1 and Example 1.

### Theorem 2

*Let* $(\Omega, \Sigma, \mu)$ *be a measure space*, $f:\Omega\rightarrow [0,1]$ *be a measurable function*, *and* *C* *be a copula*. *Then* $F:\Omega ^{2}\rightarrow[0,1]$ *defined by*

$$F(x,y):=C\bigl(f(x),f(y)\bigr) $$

*is Chebyshevian on* $\Omega^{2}$. *We also have*, *for a nonnegative integrable function* $p:\Omega\rightarrow\mathbb{R}$,

$$\begin{aligned}& \int_{\Omega}p(x)\,d\mu(x) \int_{\Omega}p(x)C\bigl(f(x),f(x)\bigr)\,d\mu(x) \\& \quad \geq \int_{\Omega} \int_{\Omega}p(x)p(y)C\bigl(f(x),f(y)\bigr)\,d\mu(x)\,d\mu(y). \end{aligned}$$

### Proof

The Chebyshevian property of *F* follows from the 2-increasing property of copulas. Therefore, we have $F(x,x)+F(y,y)\geq F(x,y)+F(y,x)$ for all $x,y \in\Omega$, or equivalently

$$C\bigl(f(x),f(x)\bigr)+C\bigl(f(y),f(y)\bigr)\geq C\bigl(f(x),f(y)\bigr)+C \bigl(f(y),f(x)\bigr). $$

Multiplying both sides by $p(x)$ and $p(y)$ and taking double integrals over $\Omega^{2}$, we have

$$\begin{aligned}& \int_{\Omega}p(x)\,d\mu(x) \int_{\Omega}p(x)C\bigl(f(x),f(x)\bigr)\,d\mu(x) \\& \quad \geq \int_{\Omega} \int_{\Omega}p(x)p(y)C\bigl(f(x),f(y)\bigr)\,d\mu(x)\,d\mu(y). \end{aligned}$$

This completes the proof. □

### Example 2

In this example, we obtain some Chebyshev type inequalities by choosing some examples of copulas. Let $(\Omega, \Sigma, \mu)$ be a measure space, $f:\Omega\rightarrow[0,1]$ be a measurable function, and $p:\Omega\rightarrow\mathbb{R}$ be a nonnegative integrable function. We have the following inequalities:

2.3 $$\begin{aligned}& \int_{\Omega} \int_{\Omega}p(x)p(y)\max\bigl\{ f(x)+f(y)-1,0\bigr\} \,d\mu(x)\,d\mu (y) \\& \quad \leq \int_{\Omega}p(x)\,d\mu(x) \int_{\Omega}p(x)\max\bigl\{ 2f(x)-1,0 \bigr\} \,d\mu(x) \\& \quad \leq \int_{\Omega}p(x)\,d\mu(x) \int_{\Omega}p(x) C\bigl(f(x),g(x)\bigr)\,d\mu(x) \\& \quad \leq \int_{\Omega}p(x)\,d\mu(x) \int_{\Omega}p(x)\min\bigl\{ f(x),g(x) \bigr\} \,d\mu(x), \end{aligned}$$

and

2.4 $$\begin{aligned}& \int_{\Omega} \int_{\Omega}p(x)p(y) \max\bigl\{ f(x)+g(y)-1,0\bigr\} \,d\mu(x)\,d\mu(y) \\& \quad \leq \int_{\Omega} \int_{\Omega}p(x)p(y) C\bigl(f(x),g(y)\bigr)\,d\mu(x)\,d\mu(y) \\& \quad \leq \int_{\Omega} \int_{\Omega}p(x)p(y)\min\bigl\{ f(x),f(y)\bigr\} \,d\mu(x)\,d\mu(y) \\& \quad \leq \int_{\Omega}p(x)\,d\mu(x) \int_{\Omega}p(x)f(x)\,d\mu(x). \end{aligned}$$

We also have the following result.

### Theorem 3

*Let* $(\Omega, \Sigma, \mu)$ *be a measure space*, $f:\Omega\rightarrow [0,1]$ *be a measurable function*. *Let* *ω* *be a positive weight on* Ω *with* $\int_{\Omega}\omega(t)\,d\mu(t)=1$. *Let* $C:[0,1]^{2} \rightarrow[0,1]$ *be a copula*. *We have the following inequalities*:

2.5 $$\begin{aligned}& \int_{\Omega}\omega C(f,f)\,d\mu+ C \biggl( f \biggl( \int_{\Omega} \omega f \,d\mu\biggr),f \biggl( \int_{\Omega}\omega f \,d\mu\biggr) \biggr) \\& \quad \geq \int_{\Omega}\omega C \biggl( f,f \biggl( \int_{\Omega}\omega f \,d\mu\biggr) \biggr)\,d\mu+ \int_{\Omega}\omega C \biggl( f \biggl( \int_{ \Omega}\omega f \,d\mu\biggr),f \biggr)\,d\mu. \end{aligned}$$

### Proof

The 2-increasing property of copulas gives us

$$C(x,x)+C(y,y)\geq C(x,y) +C(y,x) $$

for all $x,y \in[0,1]$. Take $x=f(t)$ and $y=\int_{\Omega}w(t)f(t)\, d\mu(t)$, we have

$$\begin{aligned}& C\bigl(f(t),f(t)\bigr)+C \biggl( f \biggl( \int_{\Omega}w(t)f(t)\,d\mu(t) \biggr),f \biggl( \int_{\Omega}w(t)f(t)\,d\mu(t) \biggr) \biggr) \\& \quad \geq C \biggl( f(t),f \biggl( \int_{\Omega}w(t)f(t)\,d\mu(t) \biggr) \biggr) +C \biggl( f \biggl( \int_{\Omega}w(t)f(t)\,d\mu(t) \biggr),f(t) \biggr). \end{aligned}$$

Multiplying with $\omega(t)\geq0$ and integrating over Ω give the desired result. □

In the next section, we provide further inequalities of this type.

## More inequalities

We denote the following:

$$\begin{aligned}& E_{\omega}(f) := \int_{\Omega}\omega f \,d\mu, \\& K_{\omega}(C;f,g) := \int_{\Omega} \int_{\Omega}\omega(x)\omega(y) C\bigl(f(x),g(y)\bigr)\,d\mu(x)\, d\mu(y), \\& H_{\omega}(f) := \int_{\Omega}\omega\biggl\vert f- \int_{\Omega}\omega f \,d\mu\biggr\vert \,d\mu= \int_{\Omega}\omega\bigl\vert f-E_{\omega}(f)\bigr\vert \,d\mu, \end{aligned}$$

where $\omega:\Omega\rightarrow[0,\infty)$ is *μ*-integrable with $\int_{\Omega}\omega\,d\mu=1$, $f,g:\Omega\rightarrow[0,1]$ are *μ*-measurable and $f,g\in L_{\omega}(\Omega)$, and $C:[0,1]^{2} \rightarrow[0,1]$ is a copula.

We denote by $D_{\omega}(f)$ the dispersion of a function *f* defined on a measure space $(\Omega, \Sigma, \mu)$, with respect to a positive weight *ω* on Ω with $\int_{\Omega}\omega(t)\, d\mu(t)=1$, that is,

3.1 $$ D_{\omega}(f):= \biggl( \int_{\Omega}\omega f^{2}\,d\mu- \biggl( \int_{ \Omega}\omega f \,d\mu\biggr) ^{2} \biggr) ^{\frac{1}{2}}. $$

### Theorem 4

*Let* $(\Omega, \Sigma, \mu)$ *be a measure space*, $f,g:\Omega\rightarrow [0,1]$ *be measurable functions*. *Let* *ω* *be a positive weight on* Ω *with* $\int_{\Omega}\omega(t)\,d\mu(t)=1$. *Let* $C:[0,1]^{2} \rightarrow[0,1]$ *be a copula*. *We have the following inequalities*:

$$\begin{aligned}& \bigl\vert K_{\omega}(C;f,g) -C \bigl( E_{\omega}(f), E_{\omega}(g) \bigr) \bigr\vert \\& \quad \leq \int_{\Omega} \int_{\Omega}\omega(x)\omega(y) \bigl\vert C\bigl(f(x),g(y) \bigr)-C \bigl( E_{\omega}(f), E_{\omega}(g) \bigr) \bigr\vert \, d\mu (x)\,d\mu(y) \\& \quad \leq H_{\omega}(f)+H_{\omega}(g) \leq D_{\omega}(f)+D_{\omega}(g). \end{aligned}$$

### Proof

Firstly, we have

$$\begin{aligned}& \bigl\vert K_{\omega}(C;f,g) -C \bigl( E_{\omega}(f), E_{\omega}(g) \bigr) \bigr\vert \\& \quad =\biggl\vert \int_{\Omega} \int_{\Omega}\omega(x)\omega(y) \bigl(C\bigl(f(x),g(y)\bigr)-C \bigl( E_{\omega}(f), E_{\omega}(g) \bigr) \bigr)\,d\mu(x)\,d\mu(y) \biggr\vert \\& \quad \leq \int_{\Omega} \int_{\Omega}\omega(x)\omega(y) \bigl\vert C\bigl(f(x),g(y) \bigr)-C \bigl( E_{\omega}(f), E_{\omega}(g) \bigr) \bigr\vert \, d\mu (x)\,d\mu(y). \end{aligned}$$

From the Lipschitz property of copulas, we have

$$\biggl\vert C\bigl(f(x),g(y)\bigr)-C \biggl( \int_{\Omega}\omega f \,d\mu, \int_{\Omega }\omega g \,d\mu\biggr) \biggr\vert \leq\biggl\vert f(x)- \int_{\Omega}\omega f \,d\mu\biggr\vert + \biggl\vert g(y)- \int_{\Omega}\omega g \,d\mu\biggr\vert . $$

Multiplying with $\omega(x)\omega(y)\geq0$ and integrating twice over Ω give

$$\begin{aligned}& \int_{\Omega} \int_{\Omega}\omega(x)\omega(y) \biggl\vert C\bigl(f(x),g(y) \bigr)-C \biggl( \int_{\Omega}\omega f \,d\mu, \int_{\Omega}\omega g \,d\mu\biggr) \biggr\vert \,d\mu(x)\,d\mu(y) \\& \quad \leq \int_{\Omega}\omega\biggl\vert f- \int_{\Omega}\omega f \,d\mu\biggr\vert \,d\mu+ \int_{\Omega}\omega\biggl\vert g- \int_{\Omega}\omega g \,d\mu\biggr\vert \,d\mu=H_{\omega }(f)+H_{\omega}(g). \end{aligned}$$

Finally, Schwarz’s inequality gives

$$\begin{aligned}& \biggl( \int_{\Omega}\omega\biggl\vert f- \int_{\Omega}\omega f \,d\mu\biggr\vert \,d\mu\biggr) ^{2} \\& \quad \leq \biggl( \int_{\Omega}\omega\biggl( f- \int_{\Omega}\omega f \,d\mu\biggr) ^{2}\,d\mu\biggr) \biggl( \int_{\Omega}\omega\,d\mu\biggr) \\& \quad = \int_{\Omega}\omega f^{2}\,d\mu-2 \int_{\Omega}\omega f \biggl( \int_{\Omega}\omega f \,d\mu\biggr)\,d\mu+ \int_{\Omega}\omega\biggl( \int_{\Omega}\omega f \,d\mu\biggr) ^{2}\,d\mu \\& \quad = \int_{\Omega}\omega f^{2}\,d\mu-2 \biggl( \int_{\Omega}\omega f \,d\mu\biggr) ^{2}+ \biggl( \int_{\Omega}\omega f \,d\mu\biggr) ^{2} \\& \quad = \int_{\Omega}\omega f^{2}\,d\mu- \biggl( \int_{\Omega}\omega f \,d\mu\biggr) ^{2}, \end{aligned}$$

that is,

$$\begin{aligned} \int_{\Omega}\omega\biggl\vert f- \int_{\Omega}\omega f \,d\mu\biggr\vert \,d\mu \leq&\biggl( \int_{\Omega}\omega f^{2}\,d\mu- \biggl( \int_{\Omega} \omega f \,d\mu\biggr) ^{2} \biggr) ^{\frac{1}{2}} \\ =&D_{\omega}(f). \end{aligned}$$

This completes the proof. □

### Corollary 1

*Let* $(\Omega, \Sigma, \mu)$ *be a measure space*, $f,g:\Omega\rightarrow [0,1]$ *be measurable functions*. *Let* *ω* *be a positive weight on* Ω *with* $\int_{\Omega}\omega(t)\,d\mu(t)=1$. *Let* $C:[0,1]^{2} \rightarrow[0,1]$ *be a copula*. *If* *f* *and* *g* *satisfy*

$$0\leq m_{f}\leq f\leq M_{f}\leq1, \quad \textit{and}\quad 0\leq m_{g} \leq g\leq M_{g}\leq1, $$

*then we have the inequalities*

$$\begin{aligned}& \bigl\vert K_{\omega}(C;f,g) -C \bigl( E_{\omega}(f), E_{\omega}(g) \bigr) \bigr\vert \\& \quad \leq D_{\omega}(f)+D_{\omega}(g) \\& \quad \leq \frac{1}{2}(M_{f}-m_{f})+ \frac{1}{2}(M_{g}-m_{g})\leq1. \end{aligned}$$

The proof follows from Theorem 4 and a Grüss type inequality

$$D_{\omega}(f)\leq\frac{1}{2}(M-m)\leq\frac{1}{2} $$

for *f* with the property that $0\leq m\leq f\leq M\leq1$. We omit the details.

Recall the notation

$$\begin{aligned}& E_{\omega}(f) := \int_{\Omega}\omega f \,d\mu, \\& K_{\omega}(C;f,g) := \int_{\Omega} \int_{\Omega}\omega(x)\omega(y) C\bigl(f(x),g(y)\bigr)\,d\mu(x)\, d\mu(y), \end{aligned}$$

and introduce the following notation:

$$\begin{aligned}& K_{\omega}(C;f) := \int_{\Omega} \int_{\Omega}\omega(x)\omega(y) C\bigl(f(x),f(y)\bigr)\,d\mu(x)\, d\mu(y), \\& L_{\omega}(C;f,g):= \int_{\Omega}\omega C \biggl( f, \int_{\Omega} \omega g \,d\mu\biggr)\,d\mu, \\& L_{\omega}(C,f) := \int_{\Omega}\omega C \biggl( f, \int_{\Omega} \omega f \,d\mu\biggr)\,d\mu. \end{aligned}$$

### Theorem 5

*Let* $\omega:\Omega\rightarrow[0,\infty)$ *be* *μ*-*integrable with* $\int_{\Omega}\omega\,d\mu=1$. *Let* $f,g:\Omega\rightarrow[0,1]$ *be* *μ*-*measurable and* $f,g\in L_{ \omega}(\Omega)$. *If* $C:[0,1]^{2}\rightarrow[0,1]$ *is a copula*, *then*

3.2 $$\begin{aligned} &\max\bigl\{ E_{\omega}(f)+E_{\omega}(g)-1,0 \bigr\} \leq K_{\omega }(C;f,g) \leq\min\bigl\{ E_{\omega}(f),E_{\omega}(g) \bigr\} . \end{aligned}$$

*In particular*, *we have*

3.3 $$ \max\bigl\{ 2E_{\omega}(f)-1,0 \bigr\} \leq K_{\omega}(C;f)\leq E _{\omega}(f). $$

*We also have*

3.4 $$\begin{aligned} \max\bigl\{ E_{\omega}(f)+E_{\omega}(g)-1,0 \bigr\} \leq & \int_{\Omega}\omega\max\bigl\{ f+E_{\omega}(g) -1,0 \bigr\} \,d\mu \\ \leq& L_{\omega}(C;f,g) \\ \leq& \int_{\Omega}\omega\min\bigl\{ f, E_{\omega}(g) \bigr\} \,d\mu \\ \leq& \min\bigl\{ E_{\omega}(f),E_{\omega}(g) \bigr\} . \end{aligned}$$

*In particular*,

3.5 $$\begin{aligned} \max\bigl\{ 2E_{\omega}(f)-1,0 \bigr\} \leq& \int_{\Omega}\omega\max\bigl\{ f+E_{\omega}(f)-1,0 \bigr\} \\ \leq&L_{\omega}(C,f)\leq \int_{\Omega}\omega\min\bigl\{ f,E_{ \omega}(f) \bigr\} \,d\mu \leq E_{\omega}(f). \end{aligned}$$

### Proof

We know that for any *μ*-*ω*-integrable functions *k* and *l*, we have

3.6 $$ \int_{X}\omega\min\{k,l\}\,d\mu\leq\min\biggl\{ \int_{X}\omega k \,d\mu, \int_{X}\omega l \,d\mu\biggr\} $$

and

3.7 $$ \int_{X}\omega\max\{k,l\}\,d\mu\geq\max\biggl\{ \int_{X}\omega k \,d\mu, \int_{X}\omega l \,d\mu\biggr\} . $$

Using the Fréchet-Hoeffding bounds (1.1), we obtain

3.8 $$ \max\bigl\{ f(x)+g(y)-1,0\bigr\} \leq C\bigl(f(x),g(y)\bigr) \leq \min\bigl\{ f(x),g(y)\bigr\} $$

for all $x,y\in\Omega$. If we multiply (3.8) by $w(x)w(y)\geq0$ and integrate twice over Ω, then we get

3.9 $$\begin{aligned}& \int_{\Omega} \int_{\Omega}\omega(x)\omega(y)\max\bigl\{ f(x)+g(y)-1,0 \bigr\} \, d\mu(x)\,d\mu(y) \\& \quad \leq \int_{\Omega} \int_{\Omega}\omega(x)\omega(y) C\bigl(f(x),g(y)\bigr)\,d\mu(x)\, d\mu(y) \\& \quad \leq \int_{\Omega} \int_{\Omega}\omega(x)\omega(y) \min\bigl\{ f(x),g(y) \bigr\} \,d\mu (x)\,d\mu(y). \end{aligned}$$

By (3.6) and (3.7), we get

$$\begin{aligned}& \int_{\Omega} \int_{\Omega}\omega(x)\omega(y)\min\bigl\{ f(x),g(y)\bigr\} \,d\mu (x)\,d\mu(y) \\& \quad \leq\min\biggl\{ \int_{\Omega}\omega f \,d\mu, \int_{\Omega}\omega g \,d\mu\biggr\} \end{aligned}$$

and

$$\begin{aligned}& \max\biggl\{ \int_{\Omega}\omega f \,d\mu+ \int_{\Omega}\omega g \,d\mu-1,0 \biggr\} \\& \quad \leq \int_{\Omega} \int_{\Omega}\omega(x)\omega(y) \max\bigl\{ f(x)+g(y)-1,0 \bigr\} \, d\mu(x)\,d\mu(y). \end{aligned}$$

This proves (3.2). We obtain (3.3) by setting $f\equiv g$ in (3.2).

From (1.1), we also have

3.10 $$ \max\biggl\{ f+ \int_{\Omega}\omega g \,d\mu-1,0 \biggr\} \leq C \biggl( f, \int_{\Omega}\omega g \,d\mu\biggr) \leq\min\biggl\{ f, \int_{\Omega }\omega g \,d\mu\biggr\} . $$

If we multiply (3.10) by $w\geq0$ and integrate over Ω, then we get

3.11 $$\begin{aligned} \int_{\Omega}\omega\max\biggl\{ f+ \int_{\Omega}\omega g \,d\mu-1, 0 \biggr\} \,d\mu \leq& \int_{\Omega}\omega C \biggl( f, \int_{\Omega}\omega g \,d\mu\biggr)\,d\mu \\ \leq& \int_{\Omega}\omega\min\biggl\{ f, \int_{\Omega}\omega g \,d\mu\biggr\} . \end{aligned}$$

Since

3.12 $$ \int_{\Omega}\omega\min\biggl\{ f, \int_{\Omega}\omega g \,d\mu\biggr\} \leq\min\biggl\{ \int_{\Omega}\omega f \,d\mu, \int_{\Omega}\omega g \,d\mu\biggr\} $$

and

3.13 $$ \max\biggl\{ \int_{\Omega}\omega f \,d\mu+ \int_{\Omega}\omega g \,d\mu-1,0 \biggr\} \leq \int_{\Omega}\omega\max\biggl\{ f+ \int_{\Omega }\omega g \,d\mu-1, 0 \biggr\} \,d\mu. $$

By (3.11), (3.12), and (3.13), we get (3.4). Finally, we obtain (3.5) by setting $f\equiv g$ in (3.4). □

### Lemma 2

*If* $C:[0,1]^{2} \rightarrow[0,1]$ *is a copula*, *then we have*

3.14 $$\begin{aligned} 0 \leq&\frac{1}{2}\vert u-v\vert \leq\frac{1}{2}(u+v)-C(u,v) \\ \leq&\frac{1}{2}\vert u-v\vert +\frac{1}{2}-\max\biggl\{ \biggl\vert \frac{1}{2}-u\biggr\vert , \biggl\vert \frac{1}{2}-v \biggr\vert \biggr\} \leq\frac{1}{2} \vert u-v\vert + \frac{1}{2} \end{aligned}$$

*for any* $u,v\in[0,1]$.

### Proof

Using the Fréchet-Hoeffding bounds (1.1) and the fact that

$$\min\{a,b\}=\frac{1}{2}\bigl(a+b-\vert a+b\vert \bigr),\qquad \max\{ a,b\}= \frac{1}{2}\bigl(a+b+\vert a-b\vert \bigr), $$

thus we have

$$\frac{1}{2}\bigl(u+v-1+ \vert u+v-1\vert \bigr)\leq C(u,v)\leq \frac{1}{2}\bigl(u+v-\vert u-v\vert \bigr) $$

for any $u,v\in[0,1]$. This inequality is equivalent to

3.15 $$ \frac{1}{2}\vert u-v\vert \leq \frac{1}{2}(u+v)-C(u,v) \leq\frac{1}{2}\bigl(1-\vert u+v-1\vert \bigr). $$

Applying the reverse triangle inequality, we have

$$\vert u+v-1\vert =\vert u-v+2v-1\vert =\bigl\vert u-v-(1-2v)\bigr\vert \geq \vert 1-2v\vert -\vert u-v\vert $$

for any $u,v\in[0,1]$. Similarly,

$$\vert u+v-1\vert \geq \vert 1-2u\vert -\vert u-v\vert $$

for any $u,v\in[0,1]$. Therefore,

$$-\vert u+v-1\vert \leq \vert u-v\vert -\vert 1-2v\vert , \quad \mbox{and}\quad {-}\vert u+v-1\vert \leq \vert u-v\vert -\vert 1-2u\vert , $$

giving that

$$-\vert u+v-1\vert \leq \vert u-v\vert -\max\bigl\{ \vert 1-2u\vert , \vert 1-2v\vert \bigr\} $$

for all $u,v\in[0,1]$. From (3.15), we then obtain

3.16 $$\begin{aligned} \frac{1}{2}\vert u-v\vert \leq& \frac{1}{2}(u+v)-C(u,v) \\ \leq& \frac{1}{2}+\frac{1}{2}\vert u-v\vert -\max \biggl\{ \biggl\vert \frac{1}{2}-u\biggr\vert , \biggl\vert \frac{1}{2}-v\biggr\vert \biggr\} \end{aligned}$$

for all $u,v\in[0,1]$. □

Consider the quantities

$$I_{\omega}(f,g):= \int_{\Omega} \int_{\Omega}\omega(x)\omega(y)\bigl\vert f(x)-g(y)\bigr\vert \,d\mu(x)\,d\mu(y) $$

and

$$I_{\omega}(f):= \int_{\Omega} \int_{\Omega}\omega(x)\omega(y)\bigl\vert f(x)-f(y)\bigr\vert \,d\mu(x)\,d\mu(y)=I_{\omega}(f,f). $$

By the properties of modulus, we have

$$I_{\omega}(f,g)\geq \int_{\Omega}\omega\biggl\vert f- \int_{\Omega} \omega g \,d\mu\biggr\vert \,d\mu=:H_{\omega}(f,g) $$

and

$$I_{\omega}(f)\geq \int_{\Omega}\omega\biggl\vert f- \int_{\Omega}\omega f \,d\mu\biggr\vert \,d\mu=H_{\omega}(f). $$

By Schwarz’s inequality, we also have

$$\begin{aligned} I_{\omega}(f,g) \leq& \biggl( \int_{\Omega} \int_{\Omega}\omega(x) \omega(y) \bigl(f(x)-g(x) \bigr)^{2}\,d\mu(x)\,d\mu(y) \biggr) ^{\frac{1}{2}} \\ =& \biggl( \int_{\Omega}\omega f^{2}\,d\mu-2 \int_{\Omega}\omega f \,d\mu \int_{\Omega}\omega g \,d\mu+ \int_{\Omega}\omega g^{2}\,d\mu\biggr) ^{\frac{1}{2}} \end{aligned}$$

and

$$\begin{aligned} I_{\omega}(f) \leq&\sqrt{2} \biggl( \int_{\Omega}\omega f^{2}\,d\mu- \biggl( \int_{\Omega}\omega f \,d\mu\biggr) ^{2} \biggr) ^{ \frac{1}{2}} \\ =&\sqrt{2} D_{\omega}(f). \end{aligned}$$

We have the following result.

### Theorem 6

*Let* $\omega: \Omega\rightarrow[0,\infty)$ *be* *μ*-*integrable with* $\int_{\Omega}\omega \,d\mu=1$. *Let* $f,g: \Omega\rightarrow[0,1]$ *be* *μ*-*measurable and such that* $f,g\in L_{\omega}(\Omega)$. *If* $C:[0,1]^{2}\rightarrow[0,1]$ *is a copula*, *then* (*with the notation in Theorem* 5), *we have*

3.17 $$\begin{aligned} \frac{1}{2} I_{\omega}(f,g) \leq& \frac{1}{2} \bigl( E_{\omega}(f)+ E_{\omega}(g) \bigr) -K_{\omega}(C;f,g) \\ \leq&\frac{1}{2}I_{\omega}(f,g)+\frac{1}{2}-\max\biggl\{ E_{\omega } \biggl( \biggl\vert \frac{1}{2}-f \biggr\vert \biggr),E_{\omega} \biggl( \biggl\vert \frac{1}{2}-g\biggr\vert \biggr) \biggr\} \\ \leq&\frac{1}{2} I_{\omega}(f,g)+\frac{1}{2}. \end{aligned}$$

*In particular*, *we have*

3.18 $$\begin{aligned} \frac{1}{2} I_{\omega}(f) \leq& E_{\omega}(f)-K_{\omega}(C;f) \\ \leq&\frac{1}{2}I_{\omega}(f)+\frac{1}{2}-E_{\omega} \biggl( \biggl\vert \frac{1}{2}-f \biggr\vert \biggr) \\ \leq& \frac{1}{2} I_{\omega}(f)+ \frac{1}{2}. \end{aligned}$$

*We also have*

3.19 $$\begin{aligned} \frac{1}{2} H_{\omega}(f,g) \leq& \frac{1}{2} \bigl( E_{\omega}(f)+ E_{\omega}(g) \bigr) -L_{\omega}(C;f,g) \\ \leq&\frac{1}{2}H_{\omega}(f,g)+\frac{1}{2}-\max\biggl\{ E_{\omega } \biggl( \biggl\vert \frac{1}{2}-f \biggr\vert \biggr), \biggl\vert \frac{1}{2}-E_{ \omega}(g)\biggr\vert \biggr\} \\ \leq&\frac{1}{2} H_{\omega}(f,g)+\frac{1}{2}. \end{aligned}$$

*In particular*, *we have*

3.20 $$\begin{aligned} \frac{1}{2} H_{\omega}(f) \leq&E_{\omega}(f)-L_{\omega}(C;f) \\ \leq&\frac{1}{2}H_{\omega}(f)+\frac{1}{2}-E_{\omega} \biggl( \biggl\vert \frac{1}{2}-f \biggr\vert \biggr) \leq \frac{1}{2} H_{\omega}(f)+\frac{1}{2}. \end{aligned}$$

### Proof

From Lemma 2 we have

3.21 $$\begin{aligned} \frac{1}{2} \bigl\vert f(x)-g(y)\bigr\vert \leq& \frac{1}{2}\bigl(f(x)+g(y)\bigr)-C\bigl(f(x),g(y)\bigr) \\ \leq& \frac{1}{2} \bigl\vert f(x)-g(y)\bigr\vert + \frac{1}{2}-\max\biggl\{ \biggl\vert \frac{1}{2}-f(x)\biggr\vert , \biggl\vert \frac{1}{2}-g(y)\biggr\vert \biggr\} \end{aligned}$$

for any $x,y\in\Omega$. We multiply (3.21) by $\omega(x)\omega(y)\geq0$ and integrate to get

$$\begin{aligned} \frac{1}{2}I_{\omega}(f,g) =&\frac{1}{2} \int_{\Omega} \int_{\Omega} \omega(x)\omega(y)\bigl\vert f(x)-g(y)\bigr\vert \,d\mu(x)\,d\mu(y) \\ \leq& \frac{1}{2} \biggl( \int_{\Omega}\omega f \,d\mu+ \int_{\Omega }\omega g \,d\mu\biggr) - \int_{\Omega} \int_{\Omega}\omega(x)\omega(y)C\bigl(f(x),g(y)\bigr)\,d\mu(x)\, d\mu(y) \\ \leq& \frac{1}{2} \int_{\Omega} \int_{\Omega}\omega(x)\omega(y)\bigl\vert f(x)-g(y)\bigr\vert \,d\mu(x)\,d\mu(y)+\frac{1}{2} \\ &{}- \int_{\Omega} \int_{\Omega}\omega(x)\omega(y)\max\biggl\{ \biggl\vert \frac{1}{2}-f(x)\biggr\vert , \biggl\vert \frac{1}{2}-g(y)\biggr\vert \biggr\} \,d\mu(x)\,d\mu(y) \\ \leq&\frac{1}{2}I_{\omega}(f,g)+\frac{1}{2}-\max\biggl\{ \int_{ \Omega}\omega\biggl\vert \frac{1}{2}-f\biggr\vert \,d\mu, \int_{\Omega} \omega\biggl\vert \frac{1}{2}-g\biggr\vert \,d\mu\biggr\} . \end{aligned}$$

Again, from Lemma 2 we have

3.22 $$\begin{aligned} \frac{1}{2}\biggl\vert f- \int_{\Omega}\omega g \,d\mu\biggr\vert \leq& \frac{1}{2} \biggl( f+ \int_{\Omega}\omega g \,d\mu\biggr) -C \biggl( f, \int_{\Omega}\omega g \,d\mu\biggr) \\ \leq&\frac{1}{2}\biggl\vert f- \int_{\Omega}\omega g \,d\mu\biggr\vert + \frac{1}{2}-\max \biggl\{ \biggl\vert \frac{1}{2}-f\biggr\vert ,\biggl\vert \frac{1}{2}- \int_{\Omega}\omega g \,d\mu\biggr\vert \biggr\} . \end{aligned}$$

If we multiply (3.22) by $\omega\geq0$ and integrate, then we get

$$\begin{aligned}& \frac{1}{2}H_{\omega}(f,g) \\& \quad =\frac{1}{2} \int_{\Omega}\omega\biggl\vert f- \int_{\Omega}\omega g \,d\mu\biggr\vert \,d\mu \\& \quad \leq\frac{1}{2} \biggl( \int_{\Omega}\omega f \,d\mu+ \int_{\Omega} \omega g \,d\mu\biggr) -L_{\omega}(C;f,g) \\& \quad \leq\frac{1}{2} \int_{\Omega}\omega\biggl\vert f- \int_{\Omega}\omega g \,d\mu\biggr\vert \,d\mu+\frac{1}{2}- \int_{\Omega}\omega\max\biggl\{ \biggl\vert \frac{1}{2}-f \biggr\vert , \biggl\vert \frac{1}{2}- \int_{\Omega}\omega g \,d\mu\biggr\vert \biggr\} \,d\mu \\& \quad \leq\frac{1}{2}H_{\omega}(f,g)+\frac{1}{2}-\max\biggl\{ \int_{ \Omega}\omega\biggl\vert \frac{1}{2}-f\biggr\vert \,d\mu,\biggl\vert \frac{1}{2}- \int_{\Omega}\omega g \,d\mu\biggr\vert \biggr\} \\& \quad \leq\frac{1}{2}H_{\omega}(f,g)+\frac{1}{2}. \end{aligned}$$

We obtain the particular cases by setting $f \equiv g$. □

### Remark 1

We denote the following quantities:

$$\begin{aligned}& E_{\omega} := \int_{0}^{1}t\omega(t)\,dt, \\& I_{\omega} := \int_{0}^{1} \int_{0}^{1}\omega(x)\omega(y)\vert x-y\vert \,dx \,dy, \\& H_{\omega} := \int_{0}^{1}\omega(t)\biggl\vert t- \int_{0}^{1}t\omega(t)\,dt\biggr\vert \,dt= \int_{0}^{1}\omega(t)\vert t-E_{\omega} \vert \,dt, \\& K_{\omega}(C) := \int_{0}^{1} \int_{0}^{1}\omega(x)\omega(y)C(x,y)\,dx \,dy, \\& L_{\omega}(C) := \int_{0}^{1}\omega(t)C \biggl( t, \int_{\Omega}t \omega(t)\,dt \biggr)\,dt. \end{aligned}$$

Some particular instances of interest:

- Let $\Omega=[0,1]$, $\omega:[0,1]\rightarrow[0,\infty)$, $\int_{0}^{1}\omega(t)\,dt=1$, $f(t)=g(t)=t$ $(t\in[0,1])$. Then by (3.3) we get
  $$\begin{aligned} \max\biggl\{ 2 \int_{0}^{1}t\omega(t)\,dt-1,0 \biggr\} \leq& \int_{0} ^{1} \int_{0}^{1}\omega(x)\omega(y)C(x,y)\,dx \,dy \\ =:&K_{\omega}(C) \leq \int_{0}^{1}t\omega(t)\,dt, \end{aligned}$$
  that is,
  $$\max\{2E_{\omega}-1,0\}\leq K_{\omega}(C)\leq E_{\omega}. $$
  By Theorem 6, we have
  $$\begin{aligned} \frac{1}{2}I_{\omega}\leq E_{\omega}-K_{\omega}(C)\leq \frac{1}{2}I _{\omega}+\frac{1}{2}- \int_{0}^{1}\omega(t)\biggl\vert \frac{1}{2}-t\biggr\vert \,dt\leq\frac{1}{2}I_{\omega}+ \frac{1}{2} \end{aligned}$$
  and
  $$\begin{aligned} \frac{1}{2}H_{\omega}\leq E_{\omega}-L_{\omega}(C)\leq \frac{1}{2}H _{\omega}+\frac{1}{2}- \int_{0}^{1}\omega(t)\biggl\vert \frac{1}{2}-t\biggr\vert \,dt\leq\frac{1}{2}H_{\omega}+ \frac{1}{2}. \end{aligned}$$
- Take $\Omega=[0,1]$, $\omega(t)=1$ $(t\in[0,1])$ to get
  $$\begin{aligned}& \max\biggl\{ \int_{0}^{1}f(t)\,dt+ \int_{0}^{1}g(t)\,dt-1,0 \biggr\} \\& \quad \leq \int_{0}^{1} \int_{0}^{1}C\bigl(f(x),g(y)\bigr)\,dx \,dy \\& \quad \leq \min\biggl\{ \int_{0}^{1}f(t)\,dt, \int_{0}^{1}g(t)\,dt \biggr\} . \end{aligned}$$
  When $f\equiv g$, we get
  $$\begin{aligned} \max\biggl\{ 2 \int_{0}^{1}f(t)\,dt-1,0 \biggr\} \leq \int_{0}^{1} \int_{0}^{1}C\bigl(f(x),f(y)\bigr)\,dx \,dy \leq \int_{0}^{1}f(t)\,dt. \end{aligned}$$
  By Theorem 6, we have
  $$\begin{aligned}& \frac{1}{2} \int_{\Omega} \int_{\Omega}\bigl\vert f(x)-g(y)\bigr\vert \,d\mu(x)\,d\mu(y) \\& \quad \leq \frac{1}{2} \biggl( \int_{\Omega}f \,d\mu+ \int_{\Omega}g \,d\mu\biggr) - \int_{\Omega} \int_{\Omega}C\bigl(f(x), g(y)\bigr)\,d\mu(x)\,d\mu(y) \\& \quad \leq \frac{1}{2} \int_{\Omega} \int_{\Omega}\bigl\vert f(x)-g(y)\bigr\vert \,d\mu(x)\,d\mu(y)+ \frac{1}{2}-\max\biggl\{ \int_{\Omega}\biggl\vert \frac{1}{2}-f\biggr\vert \,d\mu, \int_{\Omega}\biggl\vert \frac{1}{2}-g\biggr\vert \,d\mu \biggr\} \\& \quad \leq \frac{1}{2} \int_{\Omega} \int_{\Omega}\bigl\vert f(x)-g(y)\bigr\vert \,d\mu(x)\,d\mu(y)+ \frac{1}{2}. \end{aligned}$$
  When $f\equiv g$, we have
  $$\begin{aligned}& \frac{1}{2} \int_{\Omega} \int_{\Omega}\bigl\vert f(x)-f(y)\bigr\vert \,d\mu(x)\,d\mu(y) \\& \quad \leq \int_{\Omega}f \,d\mu- \int_{\Omega} \int_{\Omega}C\bigl(f(x), f(y)\bigr)\,d\mu(x)\,d\mu(y) \\& \quad \leq \frac{1}{2} \int_{\Omega} \int_{\Omega}\bigl\vert f(x)-f(y)\bigr\vert \,d\mu(x)\,d\mu(y)+ \frac{1}{2}- \int_{\Omega}\biggl\vert \frac{1}{2}-f\biggr\vert \,d\mu \\& \quad \leq \frac{1}{2} \int_{\Omega} \int_{\Omega}\bigl\vert f(x)-f(y)\bigr\vert \,d\mu(x)\,d\mu(y)+ \frac{1}{2}. \end{aligned}$$
  We also have
  $$\begin{aligned}& \int_{\Omega}\biggl\vert f- \int_{\Omega}g \,d\mu\biggr\vert \,d\mu \\& \quad \leq \frac{1}{2} \biggl( \int_{\Omega}f \,d\mu+ \int_{\Omega}g \,d\mu\biggr) - \int_{\Omega}C \biggl( f, \int_{\Omega}g \,d\mu\biggr)\,d\mu \\& \quad \leq \int_{\Omega}\biggl\vert f- \int_{\Omega}g \,d\mu\biggr\vert \,d\mu+ \frac{1}{2}-\max \biggl\{ \int_{\Omega}\biggl\vert \frac{1}{2}-f\biggr\vert \,d\mu, \biggl\vert \frac{1}{2}- \int_{\Omega}g \,d\mu\biggr\vert \biggr\} \\& \quad \leq \int_{\Omega}\biggl\vert f- \int_{\Omega}g \,d\mu\biggr\vert \,d\mu+ \frac{1}{2}, \end{aligned}$$
  and
  $$\begin{aligned} \int_{\Omega}\biggl\vert f- \int_{\Omega}f \,d\mu\biggr\vert \,d\mu \leq& \int_{\Omega}f \,d\mu- \int_{\Omega}C \biggl( f, \int_{\Omega}f \,d\mu\biggr)\,d\mu \\ \leq& \int_{\Omega}\biggl\vert f- \int_{\Omega}f \,d\mu\biggr\vert \,d\mu+ \frac{1}{2}- \int_{\Omega}\biggl\vert \frac{1}{2}-f\biggr\vert \,d\mu \\ \leq& \int_{\Omega}\biggl\vert f- \int_{\Omega}f \,d\mu\biggr\vert \,d\mu+ \frac{1}{2}. \end{aligned}$$
